# Minimizing proteome redundancy in the UniProt Knowledgebase

**DOI:** 10.1093/database/baw139

**Published:** 2016-12-26

**Authors:** Borisas Bursteinas, Ramona Britto, Benoit Bely, Andrea Auchincloss, Catherine Rivoire, Nicole Redaschi, Claire O'Donovan, Maria Jesus Martin

**Affiliations:** 1 European Molecular Biology Laboratory, European Bioinformatics Institute (EMBL-EBI), Wellcome Trust Genome Campus, Hinxton, Cambridge CB10 1SD, UK; 2 SIB Swiss Institute of Bioinformatics, Centre Medical Universitaire, 1 rue Michel Servet, Geneva 4 1211, Switzerland

## Abstract

Advances in high-throughput sequencing have led to an unprecedented growth in genome sequences being submitted to biological databases. In particular, the sequencing of large numbers of nearly identical bacterial genomes during infection outbreaks and for other large-scale studies has resulted in a high level of redundancy in nucleotide databases and consequently in the UniProt Knowledgebase (UniProtKB). Redundancy negatively impacts on database searches by causing slower searches, an increase in statistical bias and cumbersome result analysis. The redundancy combined with the large data volume increases the computational costs for most reuses of UniProtKB data. All of this poses challenges for effective discovery in this wealth of data. With the continuing development of sequencing technologies, it is clear that finding ways to minimize redundancy is crucial to maintaining UniProt's essential contribution to data interpretation by our users. We have developed a methodology to identify and remove highly redundant proteomes from UniProtKB. The procedure identifies redundant proteomes by performing pairwise alignments of sets of sequences for pairs of proteomes and subsequently, applies graph theory to find dominating sets that provide a set of non-redundant proteomes with a minimal loss of information. This method was implemented for bacteria in mid-2015, resulting in a removal of 50 million proteins in UniProtKB. With every new release, this procedure is used to filter new incoming proteomes, resulting in a more scalable and scientifically valuable growth of UniProtKB.

**Database URL:** http://www.uniprot.org/proteomes/

## Introduction

As the number of genomes in public nucleotide sequence databases (GenBank, ENA and DDBJ) increases, selecting the most relevant sequence record for a particular organism of interest has become increasingly complex. The UniProt Knowledgebase (UniProtKB) is the central hub for the collection of functional information on proteins (1). It has seen an exponential growth in sequences and during 2015 reached a peak of 90 million sequences. This increase, a consequence of the large number of genomes sequenced and submitted in the last few years, resulted in a high level of redundancy with many proteins over-represented in the database. Redundancy is a common problem across all organisms, but in the case of bacteria, the large number of available strains, sub-strains and isolates further exacerbates the problem. One of the major reasons for this is the increase in epidemiology studies, where different strains of the same bacterium are sequenced and submitted (2–5). For example, a search of UniProtKB in January 2015 retrieved more than 9.6 million records for *Salmonella enterica* and >6 million records for *Mycobacterium tuberculosis*, while the total number of bacterial protein records exceeded 50 million. Redundancy is a barrier to the effective use of the dataset for multiple reasons, mostly due simply to the size. Redundant sequences can hinder the discovery of novel relations between proteins and the presence of similar proteins can bias conclusions drawn from using the set (6). Removing redundant sequences is desirable to avoid highly repetitive search results for queries that closely match with an over-represented sequence (7). For example, BLAST searching with an *E. coli* protein sequence against UniProtKB would lead to thousands of identical proteins from *E. coli* being matched. It was clear that changes were needed to help users to effectively navigate the database and find meaningful biological results to further their research.

Over the years, several solutions have been attempted to deal with redundancy in biological databases. One approach aims at creating representative subsets in which no two sequences share more than a given level of identity (8). UniProt offers a subset of ‘Reference’ proteomes to help users find the most representative and best annotated set of proteins for each species. Representative Proteome Groups defined by the co-membership in UniRef clusters (9) were used to aid the selection of our Reference proteomes.

There are a host of software programs and methods aimed at minimizing redundancy within protein sequence databases. These include SkipRedundant (10), Decrease Redundancy (11), Pisces (12), UniqueProt (13), CD-HIT (14), FSA-BLAST (15), BlastClust (ftp://ftp.ncbi.nih.gov/blast/documents/blastclust.html), MinSet (16), BlastCuller (17), FastCluster (18), Leaf (6), UCLUST (19) and Compressive genomics (20). These methods differ in their use of alignment methods (local or global alignment) and clustering algorithms. It has been suggested that the proposed methods may be complementary and therefore the use of more than one software/method for removing redundancy may be desirable (21). A critical design decision in our approach was to remove redundancy at the proteome level. This decision was motivated by the fact that the majority of genome data is now being submitted as complete genomes and they are highly redundant as explained earlier. By selecting the best or the most representative, we address the challenges that redundancy poses. For those users who want every sequence no matter how redundant, we make all sequences available via the UniProt Archive (UniParc). Our method identifies redundant proteomes by performing sequence comparisons of sets of sequences for pairs of proteomes and subsequently applies graph theory to find dominating sets that provide a set of non-redundant proteomes. The pipeline to achieve this is described below.

## Methods

**Figure 1.**
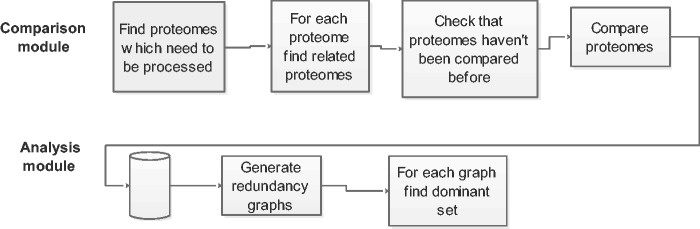
The workflow. The proteome redundancy pipeline consists of two modules: (A) Comparison and (B) redundancy removal. Each proteome is compared with every other proteome in its group. The results of the Comparison module form the input for the Redundancy removal module.

The Proteome Redundancy Minimization (PRM) pipeline consists of two modules. The first is the Proteome Comparison module which creates a graph of the relatedness between all proteomes. Second is the Redundancy Removal module that takes the proteome graph and processes it to identify the minimal set of proteomes which represent the taxonomic diversity. We describe these two modules and their algorithmic basis in the sections below and the workflow steps are schematically depicted in Figure 1.

### Proteome comparison module

Our goal for the proteome comparison module was to create a graph of all complete proteomes that represented the similarity between them. This graph is then used to identify a minimal set of proteomes. While the similarity between two proteins based on sequence alignment is a well-defined problem (22), the nature of the similarity between two proteomes is more complex. One proteome may contain proteins that have no counterpart in the other and there may be complex paralogous families where there is not a one-to-one mapping of proteins. In addition, the lengths of proteins may vary leaving us with a question of how to weigh the identity of short versus long proteins.

To ensure a scalable comparison, proteomes are compared by the identification of and comparison of sets of sequences with a minimum defined similarity threshold. Several different thresholds were tested. A 90% sequence identity cut-off was decided upon because we were interested to identify only closely related organisms and the resulting clustering fitted biological expectations. Sets of redundant proteomes were sampled at random and verified by a team of curators. A variation of the CD-HIT method, CD-HIT-2D (14), was used to identify clusters of similar sequences sharing >90% sequence identity. CD-HIT-2D is a greedy algorithm; it compares every sequence from dataset B to dataset A sequences, sorted by length, until a match over a certain threshold value is found. CD-HIT-2D was preferred over alternative clustering programs as it appeared to be the best available choice for comparing two protein datasets using each sequence from dataset A as a seed for each cluster and sequences from dataset B as members. The results of the CD-HIT-2D program were then used to calculate the proteome similarity score that takes into account the similarity of proteins and the number of unmatched sequences.

In this work, the following empirical proteome similarity score is used. The similarity of proteome B to proteome A is described as follows:

$$S(A,\;B)=\left(\sum_{b\in B}\text{length}(b)\times {\text{AS}}_{90}(A,\;b)\right)/\sum_{b\in B}\text{length}(b)\;$$

where length(b) is the length of protein b from proteome B and AS_90_(A, b) is the similarity of the alignment of protein b from proteome B with the longest sequence from proteome A, having a sequence identity of at least 90%. When there is no corresponding protein with the required sequence identity, then the value of AS equals 0.

To illustrate the use of the above score, let us take the example of two simple phage proteomes: proteome A – Enterobacteria phage MS2 and proteome B – Enteroba cteria phage MS2 (isolate ST4), where each consists of four proteins. Table 1 contains the results of CD-HIT-2D comparisons of their protein sets. The similarity calculated using the formula defined above will be (545 × 0.9853 + 393 × 0.9975 + 130 × 1.0 + 75 × 1.0)/(545 + 393 + 130 + 75) = 0.9921, which shows that proteome B is highly redundant to proteome A.

**Table 1.** Proteome similarity example^a^

Protein	Length of proteome A proteins	Length of proteome B proteins	Similarity to proteome A (%)
1	545	545	98.53
2	393	393	99.75
3	130	130	100
4	75	75	100

a This table contains the result of CD-HIT-2D comparison of two proteomes. Proteome A – Enterobacteria phage MS2 and proteome B – Enterobacteria phage MS2 (isolate ST4), each consisting of four proteins.

#### The taxonomy heuristic

Due to the large number of proteomes in UniProtKB, total pairwise comparison of proteomes is both computationally expensive and not necessary. To prune the comparison space, we found that filtering by taxonomy was very effective. Testing revealed that comparing proteomes of the same species (including strains, sub-strains and isolates) produces proteome similarities in the 0.8–1.0 range, while different species in the same genus produced proteome similarities in the range of 0.7–0.9.

#### The proteome size heuristic

To further minimize the comparison space, the size of the proteomes was compared before carrying out a full proteome comparison. The number of proteins in proteome A is compared to the number in proteome B, and if the number of proteins in A does not exceed the number of proteins in B by 10%, the full comparison is carried out. So if proteome A has 10 000 proteins and proteome B has 1000 proteins, the comparison will not be performed as 10 000 × 0.9 > 1000. The opposite comparison will be performed as 1000 × 0.9 < 10 000 (B can still be redundant to A).

The current rate of pairwise comparisons, based on the comparison strategy using the aforementioned heuristic filters, on a single modern processor server is in the range of 1500–2000 proteome comparisons per hour, or 200 proteomes a day. Parallelization of the PRM pipeline significantly improved speed, currently achieving the processing of 1000 proteomes a day on a machine with eight cores.

### Redundancy removal module

Proteome redundancy can be defined as a binary operation with the properties of non-symmetry and non-transitivity. Based on that, proteome comparisons may be represented as a directed graph, where nodes are proteomes and edges are the comparisons between them with a similarity value of at least 0.9. In this paper, we will refer to a directed graph with above-threshold comparisons as the *redundancy graph*. The problem of finding redundant/non-redundant nodes in such a graph is that of finding a minimal set of vertices that would represent the graph with a minimal loss of information. In graph theory, this is equivalent to finding dominating sets in directed graphs (23). A dominating set for a graph *G* is a subset *D* of vertices such that every vertex not in *D* is adjacent to at least one member of *D* (24). It is well known that computing a dominating set of minimal sizes is NP-hard, the best known exact algorithm runs in O(1.3247^*n*^) (25). However, for our purpose, it is sufficient to arrive at a reasonable dominating set, not necessarily the smallest as our aim is to computationally efficiently minimize redundancy.

### Algorithm

The algorithm for finding a dominating set in a directed weighted graph is an iterative process of ranking nodes in a network to find the weakest node and removing them until no further reduction is possible. The remaining nodes will be a set of non-redundant proteomes. We investigated a number of possible ways to rank proteomes in a redundancy graph. The properties described in Table 2 were considered to be of the most value and importance for ranking. Table 3 shows the example of ranking of two proteomes A and B based on parameters from Table 2. Numbers in brackets correspond to values of parameters described in Table 2 (Indegree, Outdegree, Proteome priority score, Annotation level and Previous state of redundancy).

**Table 2.** Parameters for ranking proteomes^a^

	Parameter	Description
1	Indegree	The number of proteomes found to be redundant to proteome X before elimination (the higher the better)
2	Outdegree	The number of proteomes to which proteome X was found to be redundant before elimination (the lower the better)
3	Proteome priority score	Cumulative score indicating the proteome importance Reference proteome – 2 and the other – 1
4	Annotation level	The number of entries in proteome X with curator-reviewed annotation (UniProtKB/Swiss-Prot)
5	Previous state of redundancy	A factor introduced to minimize switching between redundancy states over time. One is non-redundant and zero is redundant

a The properties described in this table were considered to be of the most value and importance for ranking proteomes in redundancy graphs.

**Table 3.** Proteome ranking example^a^

Proteome A	Proteome B	Outcome
(4,5,1,0,1)	(5,4,1,0,1)	B has higher priority
(0,5,1,0,1)	(0,3,1,0,1)	B has higher priority
(5,2,1,0,1)	(6,1,1,0,1)	B has higher priority
(5,2,1,0,1)	(5,2,2,0,1)	B has higher priority
(5,2,1,105,1)	(5,2,2,200,1)	B has higher priority

a Two proteomes A and B are ranked based on parameters from Table 2. Numbers in brackets correspond to values of parameters (Indegree, Outdegree, Proteome priority score, Annotation level and Previous state of redundancy).

**Figure 2.**
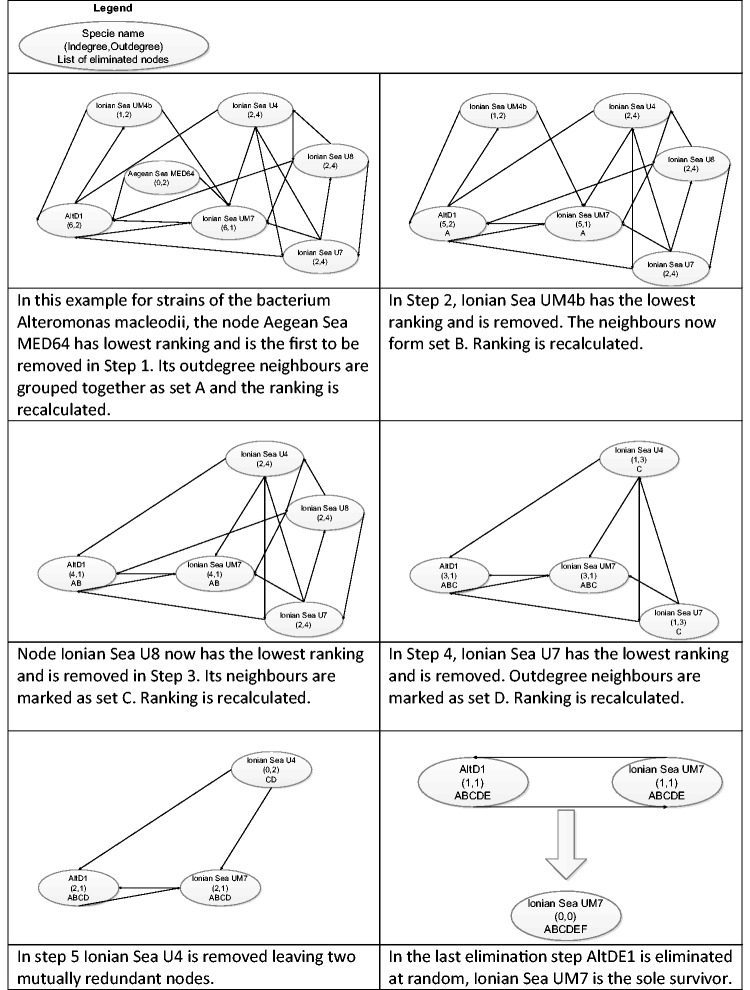
Redundancy graph reduction example. The Redundancy removal module generates redundancy graphs and finds a dominating set for each graph.

The algorithm demands that when a node is found redundant to one or more other nodes, information pertaining to the redundant node is conserved in all its outdegree neighbours. For example, if proteome A was found redundant to proteomes B and C, before A can be discarded, B and C will gain a reference to the removed node A. This specifies that at least one of B or C must remain in the final network to preserve the information contained in A. This allows further nodes to be removed in successive steps so long as nodes in the final graph summarily connect directly to all the removed nodes. The redundancy graph reduction method is described in detail using a simplified graph in Figure 2. It should be mentioned that when a proteome is eliminated, the ranking of the resulting redundancy graph is recalculated; these steps are repeated until no further elimination is possible. If two nodes have equal ranking, preference is given to the node which connects to a more comprehensive reference set of removed nodes. In the case of even ranking and information, either node may be removed at random.

Another important part of the algorithm is to define proteomes that must remain immune to the redundancy process. The criteria for the definition of UniProt Reference proteomes include all proteomes of importance to the scientific, biomedical and biotechnological communities, model organisms and representative members of other groups at phylogenetically interesting positions on the tree of life. The set of bacterial Reference proteomes was protected from redundancy; a small number of additional proteomes were added to the user request (genomes defined important by other resources).

Redundant proteomes are not discarded from the PRM module; these are available for later comparisons with new, incoming proteomes of the same taxonomic groups. A previously redundant proteome can therefore be reinstated (made non-redundant) as a consequence of new data. It should also be mentioned that proteomes flagged for redundancy are verified by curators to ensure that proteomes important to the research community remain available in the UniProt knowledgebase.

## Results and discussion

The PRM pipeline described in the previous sections was applied to UniProtKB release 2015_04 and 50 million UniProtKB records belonging to 14 856 redundant bacterial proteomes were removed. This accounted for approximately 50% of UniProtKB at that time. The PRM pipeline is now an integral part of the UniProtKB monthly production release. New incoming proteomes as well as updated proteomes are processed by the pipeline before they are imported into UniProtKB. Between releases 2015_04 and 2016_04, 61 million sequences corresponding to redundant proteomes were not integrated/deprecated from UniProtKB; these can be downloaded from the UniProt Archive (UniParc) via the Proteomes Portal.

**Figure 3.**
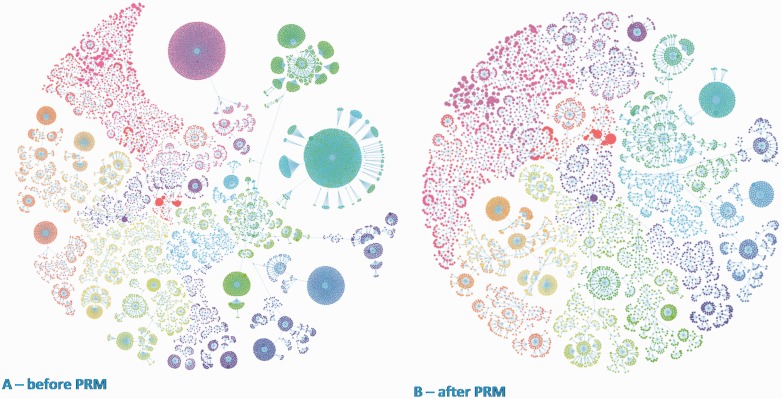
Taxonomic tree of proteomes for complete taxonomic space: before and after PRM. Proteomes were mapped before and after PRM taking into account the taxonomic relationships between the proteomes (panels A and B). Each node corresponds to a proteome, while a cluster represents a species grouping. The cluster size indicates the number of proteomes available for a particular species. The effect of PRM is clearly visible in the finer grained figure to the right (panel B).

**Figure 4.**
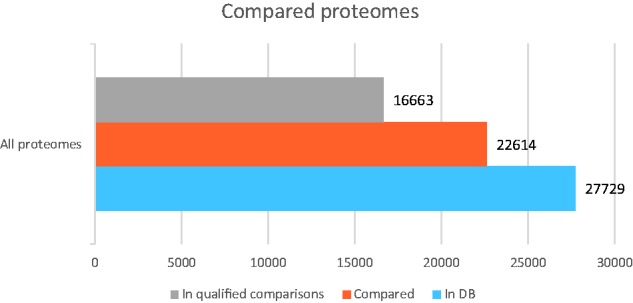
Compared proteomes. Nearly 16 663 proteomes (60% of the total) appeared in above-threshold comparisons, i.e. at least 90% similar to another proteome of the same taxonomic group.

**Figure 5.**
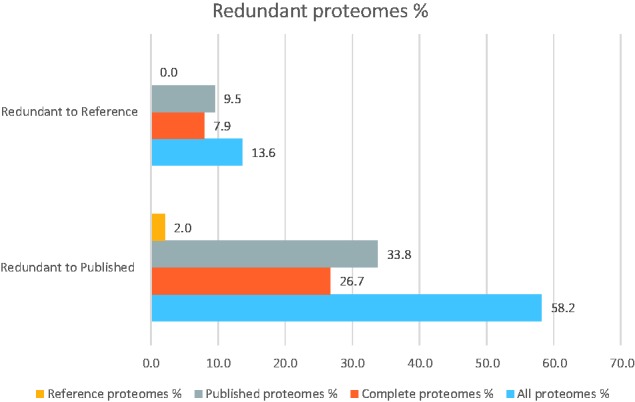
Percentages of redundant proteomes. The distribution of redundant proteomes is shown as a fraction of the Reference (13.65%) and all proteomes (58.2%), respectively.

To visualize the impact of the PRM pipeline on the database, we have mapped the proteomes before and after, taking into account the taxonomic relationships between the proteomes for all the taxonomic space (Figure 3A). Each node corresponds to a proteome, while a cluster represents a species grouping. The sizes of clusters indicate the number of proteomes available for a particular species. The effect of the PRM pipeline is clearly visible in the figure to the right (Figure 3B). Figures 4 and 5 highlight the extent of proteome redundancy in UniProtKB for all taxonomy before data reduction (i.e. release 2015_03). Nearly 16 663 proteomes (60% of the total) appeared in above-threshold comparisons, i.e. at least 90% similar to another proteome of the same taxonomic group. Figure 5 shows the distribution of redundant proteomes as a fraction of the Reference proteomes (13.6%) and all proteomes (58.2%), respectively. It was found that 6635 proteomes (roughly 29%) had no siblings (with a comparable number of proteins) within the species branch, which indicates the uniqueness of those proteomes. The remaining proteomes (16 231: 71%) were in at least one above-threshold comparison. Nearly 55% of proteomes from the bacterial kingdom were redundant and removed from UniProtKB. These redundant bacterial proteomes accounted for approximately 50 million UniProtKB records. Table 4 summarizes the results of redundancy analysis of four frequently sequenced, highly redundant bacterial species. It clearly shows that an increase in proteomes in graphs does not lead to a similar increase in non-redundant proteomes. Further analysis revealed that the number of non-redundant proteomes correlates with the number of processed taxonomic branches regardless of the total number of proteomes within each species' taxonomic branches (Table 4). This would indicate a more linear growth trend for UniProtKB in the coming releases and one may even be able to foresee a point where all taxonomic branches are represented and growth levels out.

**Table 4.** Measurable reduction in proteome redundancy^a^

Metric name	*Escherichia coli*	*Staphylococcus aureus*	*Mycobacterium tuberculosis*	*Bordetella pertussis*
Total proteomes	1987	4130	1705	37
Redundant proteomes	1922	4115	1697	34
Non-redundant proteomes	65	15	8	3
Obsolete UniProtKB records	9 642 498	11 015 716	6 779 520	133 038
Retained UniProtKB sequences	317 761	41 068	32 028	10 473

a The number of non-redundant proteomes correlates with the number of processed taxonomic branches regardless of the total number of proteomes within each taxonomic branch.

### The proteomes portal

**Figure 6.**
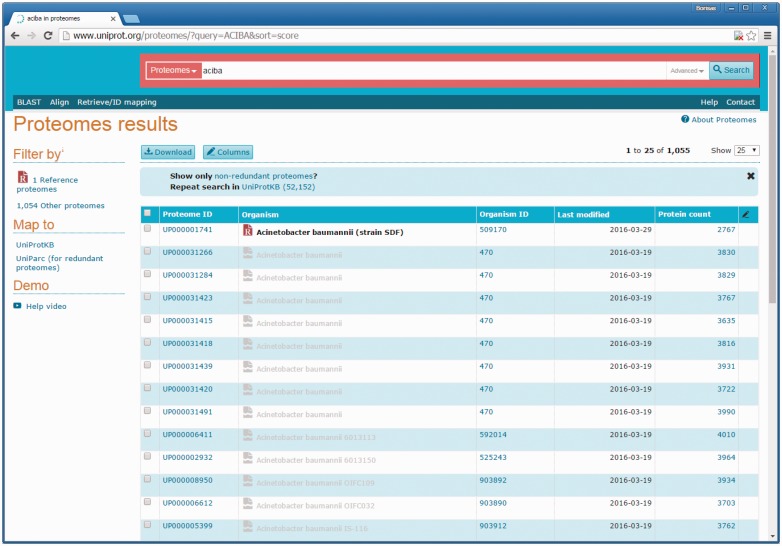
UniProt Proteome search results. Querying by species names, taxonomy identifiers, organism codes as well as the newly introduced proteome identifiers is supported. When querying by species, one or more Reference proteomes are indicated at the top of the list followed by any other proteomes available. Redundant proteomes appear in grey and users are directed to an alternate non-redundant proteome available for the species.

**Figure 7.**
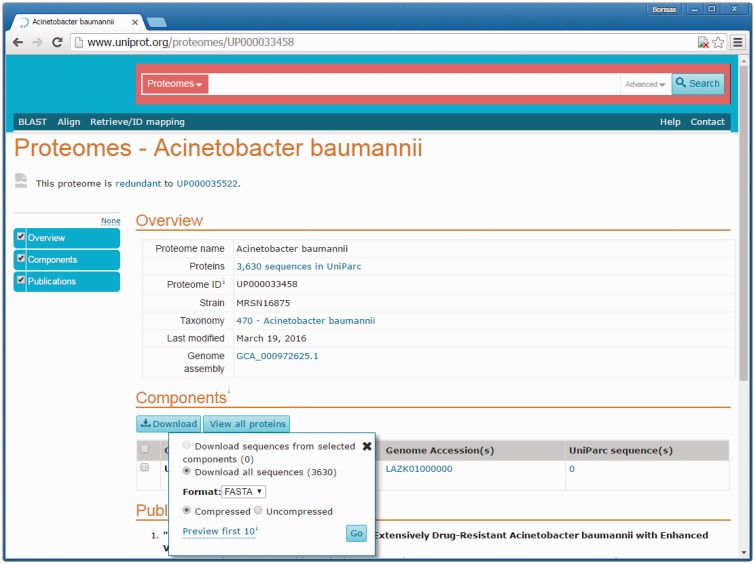
Proteome page with opened download window. This figure shows the proteome page for redundant proteomes with opened download window. The sequences in UniParc can be downloaded in a range of formats using that download window.

The UniProtKB Proteomes portal (http://www.uniprot.org/proteomes) was launched in spring 2015 to enable users to access the proteomes available for different taxonomic groupings. Querying by species name, taxonomy identifier, organism codes as well as the newly introduced proteome identifiers is supported (Figure 6). When queried by species, one or more of the related Reference proteomes are indicated at the top of the list followed by any other proteomes available. With proteome redundancy removal, a major improvement in the representation of relevant data was achieved. A unique proteome identifier groups together the set of components that make up each proteome. In the majority of cases, the proteome ID corresponds to a single assembly of a completely sequenced genome. Redundant proteomes appear in grey on these pages. The sequences corresponding to redundant proteomes can be downloaded from UniParc. Figure 7 shows a proteome page for an *Acinetobacter baumannii* redundant proteome with an opened download window. Sequences in UniParc can be downloaded in a range of formats via the download window. Most usefully, users are also directed to an alternate non-redundant proteome available for the species. From the search results, users can navigate to an individual proteome page containing a short description of the organism and sequencing project (for Reference proteomes), genome assembly, components and submission references. The Proteome Page interface (Figure 6) also facilitates the download of multiple proteomes for comparative analyses, by ticking checkboxes next to proteomes of interest (such as a reference proteome and its redundant proteomes for example) and subsequently clicking the Download button above the result list.

## Future work

As sequencing projects continue to increase in scale, we will extend proteome redundancy minimization to other taxonomic branches as needed. For example, fungi are suitable candidates for PRM as there is an increasing level of redundancy within this clade. However, several challenges have to be overcome before this can be implemented in other major branches such as non-fungal eukaryotes and viruses. Viral proteomes are particularly sensitive to changes due to their very small genomes. The number of viral proteomes in individual clusters by far exceeds those of bacteria and this can pose a challenge for all-against-all comparisons. Therefore, viruses might require different heuristics or a different approach altogether. Non-fungal eukaryotes, on the other hand, do not suffer from an abundance of proteomes for the same species as yet. However this is also likely to change as the sequencing revolution continues.

We will continue to find ways of improving the search and the usability of proteomics data via the Proteome portal data and very much welcome feedback from the community in both its usability and suggestions for further Reference proteomes.

## Funding

This work was supported by the National Institutes of Health [U41HG007822, U41HG002273, R01GM080646, P20GM103446, U01GM120953]; Swiss Federal Government through the State Secretariat for Education, Research and Innovation and European Molecular Biology Laboratory core funds. Funding for open access charge: National Institutes of Health [U41HG007822].

*Conflict of interest*. None declared.
